# CLEFMA Induces the Apoptosis of Oral Squamous Carcinoma Cells through the Regulation of the P38/HO-1 Signalling Pathway

**DOI:** 10.3390/cancers14225519

**Published:** 2022-11-10

**Authors:** Pei-Ni Chen, Chiao-Wen Lin, Shun-Fa Yang, Yu-Chao Chang

**Affiliations:** 1 Institute of Medicine, Chung Shan Medical University, Taichung 402, Taiwan; 2 Department of Medical Research, Chung Shan Medical University Hospital, Taichung 402, Taiwan; 3 Institute of Oral Sciences, Chung Shan Medical University, Taichung 402, Taiwan; 4 Department of Dentistry, Chung Shan Medical University Hospital, Taichung 402, Taiwan; 5 School of Dentistry, Chung Shan Medical University, Taichung 402, Taiwan

**Keywords:** CLEFMA, p38, HO-1, apoptosis, oral squamous carcinoma

## Abstract

**Simple Summary:**

Oral squamous cell carcinoma (OSCC) constitutes more than 90% of head and neck cancers and a high prevalence rate in some parts of the world. Alcohol consumption, use of snuff, and several other factors including genetic makeup are associated with 90% of patients with oral cancer. However, the molecular mechanisms involved in CLEFMA-mediated apoptotic cell death of human OSCC remain poorly understood. Our study found that CLEFMA induced the heme oxygenase-1 (HO-1) level by activating p38 mitogen-activated protein kinase signalling cascade and subsequently activated caspase-dependent cell death, which is critical to the anti-cancer effect in OSCC.

**Abstract:**

The purpose of this research was to evaluate the impact and the underlying molecular mechanism of CLEFMA-induced cell death in human OSCC. The anti-tumour properties of CLEFMA in oral cancer were explored using colony formation, flow cytometry, human apoptosis array, Western blot, and immunohistochemistry assays. The in vivo anti-tumour effect of CLEFMA administered by oral gavage was evaluated using SCC-9-derived xenograft-bearing nude mouse models. CLEFMA significantly suppressed colony formation and elicited cellular apoptosis in oral cancer cells. CLEFMA treatment remarkably increased phosphorylated p38 and HO-1 along with cleavage of poly ADP-ribose polymerase and activation of caspase-8, -9, and -3 in HSC-3 and SCC-9 cells. Administration of HO-1 small interfering RNA significantly protected the cells from CLEFMA-induced caspase-3, -8, and -9 activation. Attenuation of p38 activity by the pharmacologic inhibitor SB203580 dramatically reduced CLEFMA-induced caspase-3, -8, and -9 activation and HO-1 expression in OSCC. The subcutaneous murine xenograft models showed that CLEFMA in vivo suppressed tumour growth in implanted SCC-9 cells. All of these findings indicated that CLEFMA induced apoptosis through the p38-dependent rise in HO-1 signal transduction cascades in OSCC.

## 1. Introduction

Drugs that act on the apoptotic machinery form one of the major treatments for cancer management and are characterised by morphological alterations, including extensive plasma membrane blebbing, condensation of chromatin, endonucleolytic cleavage of chromosomal DNA, increased mitochondrial membrane permeability, decreased Δ*Ψ*m, and caspase activation [1]. Caspases are initially synthesised within cells as inactive pro-caspases and must become active cleaved caspases during apoptosis. To undergo apoptosis, effector and initiator caspases are activated by extrinsic stimuli through death receptors or intrinsic stimuli that lead to mitochondrial depolarisation [2]. The stress-responsive MAPKs are threonine and serine protein kinases, including JNK, p38, and ERK, and have been implied in many aspects of cell survival and apoptotic cell death regulation in various cancer cells [3,4,5]. The dysregulation of apoptosis is a key driver of tumourigenesis, and anti-tumour strategies in clinical cancer therapy focus on targeting apoptotic pathways [6]. However, a lack of apoptotic cell death may result in drug interactions or off-target effects. Therefore, a goal and mainstay of clinical oncology in oral squamous cell carcinoma, which is a primary cause of mortality and morbidity worldwide, is the development of molecular targeted therapy for the regulation of apoptosis.

Phytochemicals have been broadly investigated in the clinical or preclinical trials of chemopreventive potentials, medicative drugs, or therapeutic aspects for anti-tumour research [5,7,8]. Among the well-studied phytochemicals, 4-[3,5-Bis(2-chlorobenzylidene)-4-oxo-piperidine-1-yl] 4-oxo-2-butenoic acid (CLEFMA), a structural analogue of curcumin, has pharmacological activities for clinical conditions, such as inflammation [9], shock-associated gut injury [10], and cancer [11,12]. CLEFMA treatment exerted effects that inhibited haemorrhagic shock-induced gut dysfunction, proteasome activity, and 20S substrate ornithine decarboxylase degradation in a hypovolemic shock rat model [10]. The treatment of A549 cells with CLEFMA improved cisplatin efficacy and decreased cisplatin ototoxicity by regulating apoptosis-inducing factor and caspase-12 [12]. CLEFMA elicits apoptotic cell death by activating intrinsic caspase 9 and extrinsic caspase 8 initiators and targeting the JNK and p38 signalling cascades in human osteosarcoma HOS and U2OS cells [11]. CLEFMA possesses anti-cancer activities including reduction of cell growth, increase in cell-killing efficacy, and stimulation of autophagic cell death in H441 cells [13]. CLEFMA exhibits anti-metastatic and proapoptotic activities against osteosarcoma and lung cancer. However, the anti-cancer effects of CLEFMA on OSCC and the targeted molecular signalling pathways of such effects are not thoroughly established. The purpose of the present study is to further delineate the involvement of the p38/HO-1 signal transduction cascades by treatment of CLEFMA in human OSCC cell lines. This study provides the signalling mechanisms for the proapoptotic activities of CLEFMA and suggests its potential contribution and scope in the clinical development of treatment of human OSCC.

## 2. Materials and Methods

### 2.1. Cell Culture

Human tongue carcinoma cell lines HSC-3 (tumours of metastatic lymph nodes) and SCC-9 (primary OSCC) were acquired from Merck KGaA (SCC193, Darmstadt, Germany) and ATCC (CRL-1629, Manassas, VA, USA), respectively. SCC-9 was incubated in DMEM/F12 supplemented 10% foetal bovine serum (FBS). HSC-3 was cultured in DMEM-high glucose (SLM-120-B, Merck KGaA, Darmstadt, Germany) containing 10% FBS. Smulow–Glickman (SG) cells (human gingival epithelial cells) were originally derived from human gingiva and were cultured in DMEM supplemented with 10% FBS [14].

### 2.2. MTT Cell Viability Assay

The SCC-9 and HSC-3 cells were seeded at a density of 6 × 10^4^ cells/well in 24-well plates, incubated overnight, and exposed to 0.1% DMSO (control group) or different concentrations of CLEFMA (0–16 μM) for 24 h. After treatment of CLEFMA, the cells were rinsed with PBS and incubated with a culture medium containing of MTT reagent (0.5 mg/mL; Millipore Sigma, St. Louis, MI, USA) for 3 h. Then, 1 mL of isopropanol was added to dissolve the MTT formazan crystals in viable cells and they were observed spectrophotometrically at 570 nm [15].

### 2.3. Colony Formation Assay

Colony formation assay is a method to evaluate the cell survival and growth based on the capacity of single cells to grow into colonies. Three thousand HSC-3 and SCC-9 cells were seeded into a six-well plate and exposed to 0.1% DMSO (control group) or CLEFMA (0, 1, 2, 4, and 8 μM) for 10 days. After incubation, the cells were stained with crystal violet and the colony number was calculated [16].

### 2.4. Sub-G1 Phase Ratio Analysis

SCC-9 and HSC-3 were cultured with 0–8 µM CLEFMA for 24 h and then incubated with 0.25% trypsin-EDTA solution, collected, and fixed in cold 70% ethanol for 24 h. The fixed cells were suspended with propidium iodide (PI) buffer at room temperature for 30 min in the dark. A BD flow cytometer system was used to analyse the distribution of the cell cycle and Cell Quest software was used to quantitate the flow cytometry data [17].

### 2.5. Apoptotic Cell Death Assay

Apoptotic cell death was measured with BD Annexin V-FITC Apoptosis Detection Kit (BD Bioscience, Becton Dickinson Co., Franklin Lakes, NJ, USA). Cells were seeded in 10 cm dishes and exposed to 0–8 µM CLEFMA for 24 h. The cells were harvested, resuspended, and incubated with binding buffer at room temperature. After staining, the late apoptotic and early apoptotic cells were examined through FACS Calibur flow cytometry performed using CellQuest software [18].

### 2.6. Proteome Profiler Human Apoptosis Array

The relative levels of 35 different human apoptosis-related proteins were determined using a R&D proteome profiler human apoptosis array kit (ARY009). HSC-3 was incubated with or without CLEFMA for 24 h and rinsed with PBS. After treatment, the cells were solubilised at 1 × 10^7^ cells/mL in lysis buffer on ice. The whole-cell lysates for the array with 350 µg of protein were examined according to the guidelines of the kit’s manufacturers. Dot blots were visualized by exposure to an X-ray film and photographed and detected with a Bio-Rad, Hercules, CA, USA Molecular Imager Gel Doc XR system [19].

### 2.7. Western Blot Analysis

HSC-3 and SCC-9 cells were exposed to 0.1% DMSO (control group) or different concentrations of CLEFMA for 24 h. Protein lysates were prepared with a GE protein extraction buffer kit supplemented protease inhibitor cocktails (Millipore Sigma, St. Louis, MI, USA) for 20 min. After the cell debris was removed, protein concentration was observed through Bio-Rad’s Bradford assays. Thirty micrograms of protein was separated in 10–12.5% SDS-PAGE and transferred onto PVDF transfer membranes. The transferred PVDF membranes were incubated with StartingBlock blocking buffer (Thermo Fisher Scientific, Waltham, MA, USA) and then probed with primary antibodies for 18 h. The PVDF membranes were reacted with specific secondary antibodies. An enhanced chemiluminescence kit (Thermo Fisher Scientific, Waltham, MA, USA) was added to the PVDF membranes and the chemiluminescence signal was visualised and measured using a CCD camera system (ImageQuant LAS 4000 Mini) [20].

### 2.8. Detection of Active Caspase-3

Intracellular activities of caspase-3 were measured with fluorescence microscopy. A BioTracker NucView 488 green caspase-3 dye (Merck KGaA, Sigma-Aldrich, St. Louis, MI, USA) was used. The cells were cultured with CLEFMA (0–8 µM) and fluorescence reagent (2 μM) for 24 h. Afterward, the cells were analysed through fluorescence microscope with the green channel filter.

### 2.9. Small Interfering (Si) RNA Transfection

*HMOX1* gene (heme oxygenase-1, HO-1) silencing was performed using the Silencer Select siRNA targeting *HMOX1* (#4390824, Ambion, Thermo Fisher Scientific, WinsFord, UK) and a non-targeting siRNA (NT) (negative control siRNA, #4390844, Ambion, Thermo Fisher Scientific, WinsFord, UK). In brief, cells were transfected with *HMOX1* siRNAs through Invitrogen transfection reagent for siRNA (150 pmol) delivery.

### 2.10. Tumour Growth in Nude Mice Model

The animal research protocol was approved by the IACUC (approval number: 2156). Four-week-old male severe combined immunodeficiency nude mice were obtained from National Laboratory Animal Center (Taipei, Taiwan). For tumour growth assay, SCC-9 cells (2.5 × 10^6^ cells in 0.1 mL of PBS per mouse) were injected into the right front axilla of the mice by subcutaneous administration. After 9 days, the mice were divided into three groups of five animals and the vehicle (0.22% polyethylene glycol 400 sterile water solution; five times a week; control group), 0.2 mg/kg CLEFMA, and 0.4 mg/kg CLEFMA (CLEFMA was dissolved in 0.22% polyethylene glycol 400; five times a week) were orally administered to nude mice. Tumour growth was detected every 3 days during the study and measured with vernier calipers. After 27 days, the primary tumour tissues were collected, weighed, and then fixed in 3% paraformaldehyde, and paraffin-embedded slides were probed with anti-Ki67 antibodies by immunohistochemistry analysis [21].

### 2.11. Statistical Analysis

Student’s t-test (Sigma-Stat 2.0, San Jose, CA, USA) was used to calculate and determine the significant differences. *p* value < 0.05 was indicated statistically significant. The values involve the means ± standard deviation (SD) at three independent experiments.

## 3. Results

### 3.1. Decline in Cell Viability by CLEFMA in OSCC

**Figure 1:**
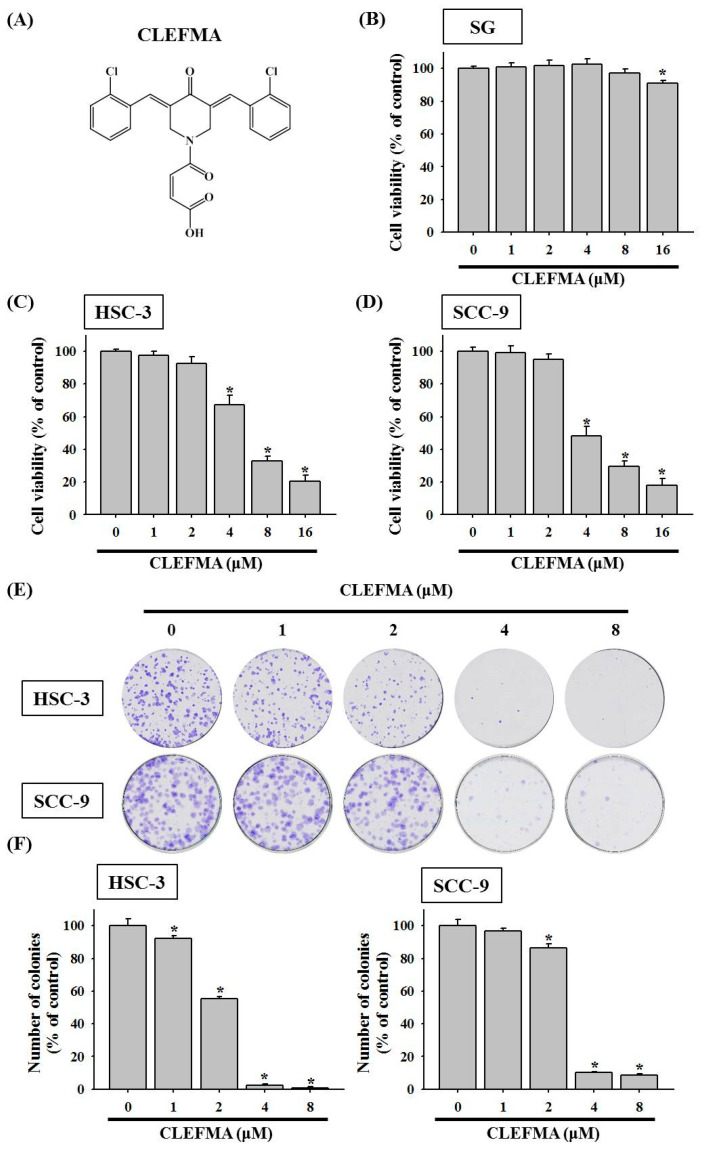

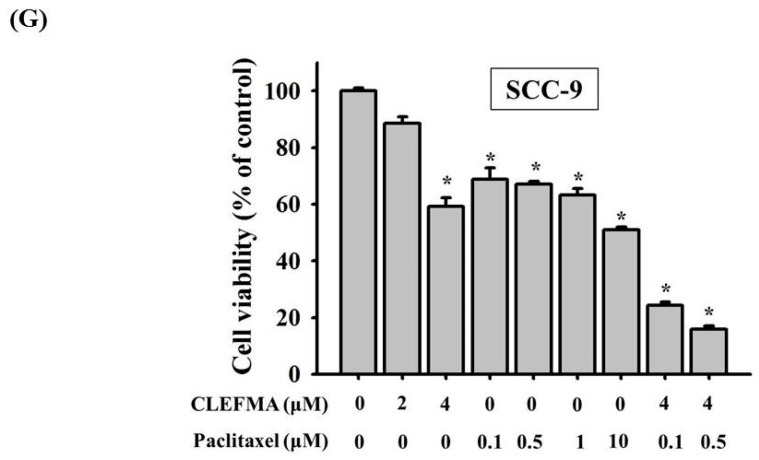
Inhibitory effects of CLEFMA on viability and formed cell colonies in human OSCC cells. (**A**) The molecular structure of CLEFMA. (**B**) SG cells, (**C**) HSC-3, and (**D**) SCC-9 cells treated with 0–16 μM CLEFMA for 24 h and then subjected to MTT cell viability assay. (**E**) Digital image showing colonies produced by HSC-3 and SCC-9 cells, treated with 0–8 μM CLEFMA with colony formation assay. (**F**) The quantification of formed cell colonies. (**G**) SCC-9 cells were treated with paclitaxel (taxol) alone or in combination with CLEFMA and then subjected to MTT assay. Data represent the average ± SD from three independent experiments. * *p* < 0.05 compared with the DMSO control group.

The molecular structure of CLEFMA is shown (Figure 1A). To examine the anti-cancer activity of CLEFMA on OSCC, the change in cell viability in response to CLEFMA was evaluated. Moreover, the effect of CLEFMA on the cell viability and cytotoxicity of non-malignant human gingival epithelial cell SG cell lines was also determined. The effects of 24 h treatment with CLEFMA at 0–16 μM on the cell viability of human gingival epithelial cell SG cell lines, OSCC lines HSC-3 (tumours of metastatic lymph nodes), and SCC-9 (primary OSCC) were analysed by MTT assay. After 24 h treatment, the cell viability of non-malignant SG cells in the presence of concentrations of CLEFMA (0–8 µM) was not significantly different compared with DMSO vehicle (control group), but slightly reduced with 16 μM CLEFMA treatment (Figure 1B). However, CLEFMA remarkably suppressed the cell viability of human OSCC after 24 h of treatment compared with the 0.1% DMSO vehicle (Figure 1C,D). The IC50 of CLEFMA in HSC-3 following 24 h of treatment was 6.122 μM, while the IC50 of CLEFMA in SCC-9 cells was 4.808 μM. CLEFMA at 4 μM remarkably reduced the viability of HSC-3 (Figure 1C) and SCC-9 cells (Figure 1D). Next, to explore the treatment of CLEFMA on the long-term cell growth of OSCC, we tested the effect of CLEFMA on clonogenic growth using the colony formation assay. The colony formation assay results exhibited that CLEFMA greatly inhibited the clonogenic proliferation of HSC-3 and SCC-9 cells after 10 days of incubation (Figure 1E). The long-term growth of HSC-3 and SCC-9 was dramatically diminished after 4 and 8 μM CLEFMA treatment compared with the DMSO control, respectively (Figure 1E). CLEFMA at 1 μM remarkably declined the colony formation of HSC-3 cells, while the colony formation of SCC-9 was significantly attenuated at 2 μM CLEFMA (Figure 1F). Moreover, SCC-9 cells were incubated with different doses of paclitaxel (taxol; clinical chemotherapy drug) at 0–10 μM alone or in combination with CLEFMA (4 μM) to evaluate the effect of CLEFMA treatment on the anti-cancer activity of paclitaxel by MTT assay for 24 h. The percentages of viability inhibition reduced to 50.98% in the 10 μM paclitaxel treatment of SCC-9 cells compared with the DMSO vehicle, respectively (Figure 1G). SCC-9 treated with a combination of paclitaxel (0.1–0.5 μM) and 4 μM CLEFMA for 24 h reduced the viability of SCC-9 cells to the highest extent (Figure 1G). Our finding revealed that CLEFMA could improve the inhibitory effect of paclitaxel on cell viability.

### 3.2. Increase in Apoptotic Cell Distribution by CLEFMA in OSCC

**Figure 2:**
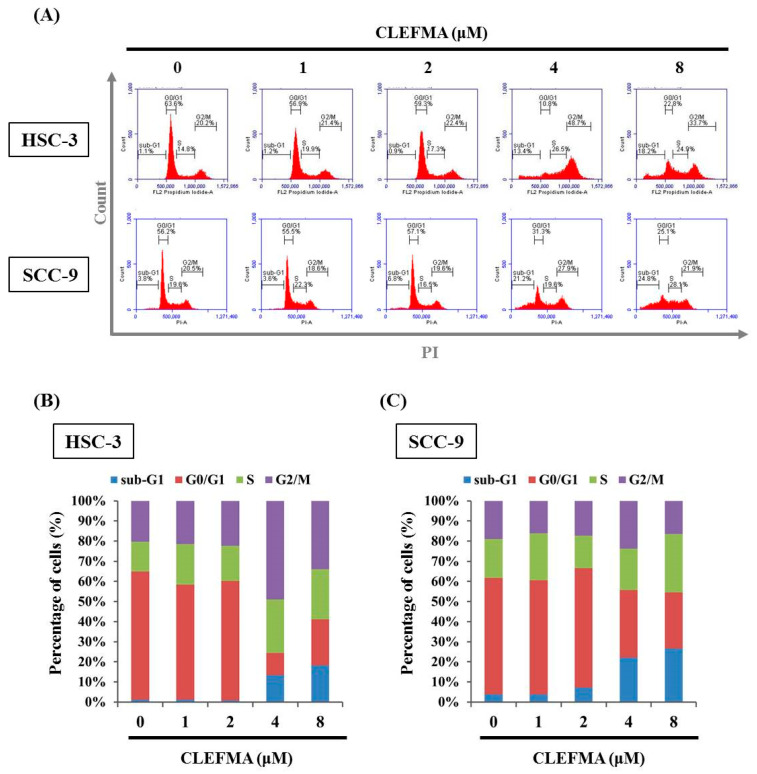
CLEFMA increased the sub-G1 phase ratio in human OSCC. (**A**) HSC-3 and SCC-9 cells were incubated with CLEFMA at indicated concentrations for 24 h and then incubated with a PI buffer. After staining, the cells were subjected to cell cycle distribution analysis via flow cytometry. The quantification results of the cell cycle population of (**B**) HSC-3 and (**C**) SCC-9 cells are shown in the bar graph. Quantification analysis was acquired from three independent experiments and expressed as mean ± SD.

Given that CLEFMA reduced the cell viability of OSCC, whether CLEFMA induced apoptosis of SCC-9 and HSC-3 cells was determined. The impact of CLEFMA on cell cycle state was examined via PI staining by flow cytometry. After 24 h treatment with CLEFMA, CLEFMA significantly increased the sub-G1 phase ratio compared with the 0.1% DMSO control in the HSC-3 and SCC-9 cells (Figure 2A). The apoptotic cell proportion of the cells in the sub-G1 phase clearly increased from 1.1% (0.1% DMSO control group) to 18.2% (*p* < 0.05, 8 μM CLEFMA group) and from 3.8% (0.1% DMSO control) to 24.8% (*p* < 0.05, 8 μM CLEFMA group) in the 8 μM CLEFMA group of HSC-3 (Figure 2B) and SCC-9 (Figure 2C).

### 3.3. Apoptotic Effect of CLEFMA in OSCC

**Figure 3:**
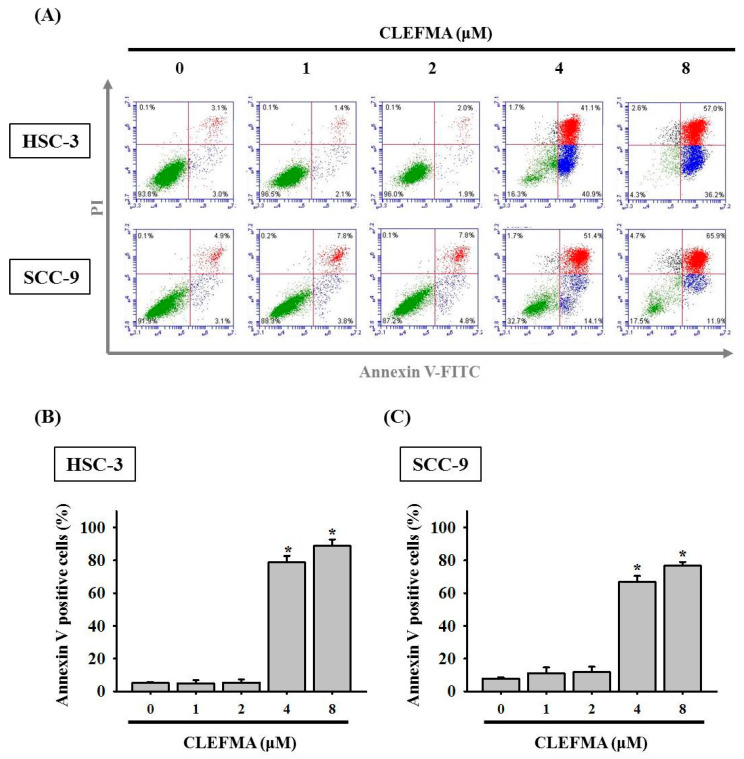
CLEFMA induced apoptosis in OSCC. (**A**) Cells were treated with various CLEFMA concentrations and then subjected to apoptosis assay through flow cytometry with annexin V/PI staining for 24 h. The quantification results of apoptosis assay of (**B**) HSC-3 and (**C**) SCC-9 cells are shown in the bar graph. Data represent the average ± standard deviation (SD) from three independent experiments. * *p* < 0.05 compared with the DMSO control group.

Annexin V-fluorescein isothiocyanate (FITC)/PI double-staining results showed that early apoptotic cell death (PI-negative and annexin V-positive cells in the lower right quadrant) and end-stage (late) apoptotic cell death (annexin V/PI-positive cells in the upper right quadrant) all dramatically increased after treatment with 4–8 μM CLEFMA in HSC-3 and SCC-9 cells (Figure 3A). The percentages of total apoptotic cells significantly increased to 88.9% and 76.9% in the 8 μM CLEFMA group of HSC-3 (Figure 3B) and SCC-9 (Figure 3C) compared with the DMSO control, respectively. The results showed that the pro-apoptotic effects of CLEFMA are more potent on HSC-3 than on SCC-9 cells.

### 3.4. Regulation of cIAP-1 and HO-1 by CLEFMA-Induced Caspase-Mediated Apoptosis in OSCC

**Figure 4:**
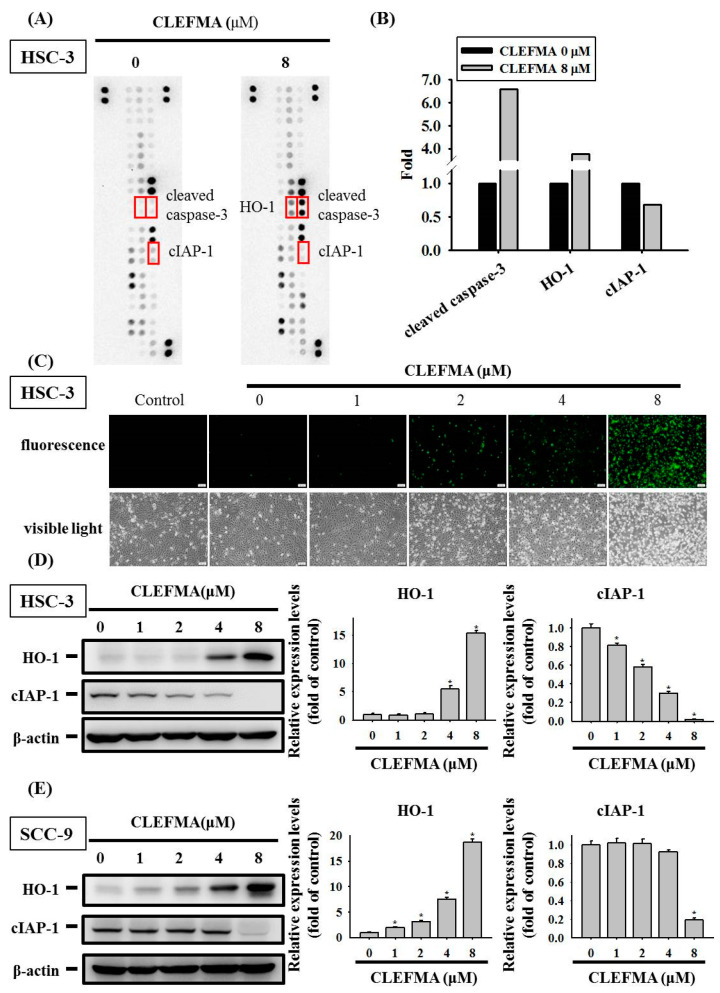
Elevated effects of CLEFMA on caspase-3 activation and HO-1 expression in human OSCC cells. (**A**) HSC-3 cells were incubated for 24 h with 8 μM CLEFMA and compared with untreated lysate (DMSO control group), and the cell lysates were used to detect 35 different apoptosis-related proteases using the human proteome profiler apoptosis array. (**B**) The quantification of cleaved caspase-3, HO-1, and cIAP-1 from human apoptosis array. (**C**) Live cell imaging apoptosis dye for caspase-3 activity used to detect apoptotic death using fluorescence microscopy with the corresponding filter (green channel). Western blot was performed on HO-1 and cIAP-1 using β-actin as the internal control in (**D**) HSC-3 and (E) SCC-9 cells. Quantitative data were acquired from three independent experiments and expressed as mean ± SD. * represent *p* < 0.05 as compared with the DMSO vehicle.

**Figure 5:**
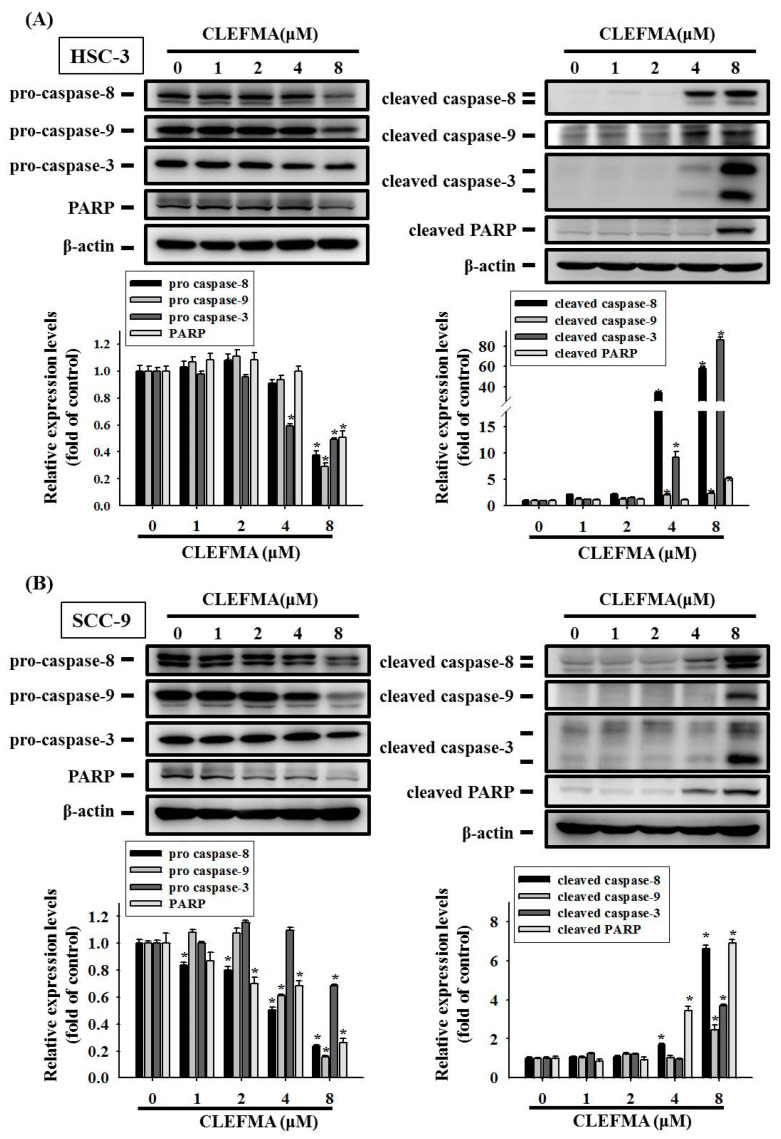
Apoptotic patterns of OSCC cells treated with CLEFMA. (**A**) HSC-3 and (**B**) SCC-9 cells were treated with 0–8 μM CLEFMA. Western blot analysis was performed. β-actin was used as a loading control. Proteins signals were visualised with an ECL imaging system. Quantitative analyses were obtained from three independent experiments and expressed as mean ± SD. * represent *p* < 0.05 as compared with the control.

Given that CLEFMA significantly increases the apoptotic cell death of OSCC, a proteome profiler human apoptosis array was used in screening the expression levels of 35 apoptotic proteins. CLEFMA reduced the protein levels of the cIAP-1, whereas the expression levels of HO-1 and cleaved caspase-3 were elevated in HSC-3 cells incubated with 8 µM CLEFMA (Figure 4A). Quantitative analyses by human apoptosis array revealed that CLEFMA increased the levels of cleaved caspase-3 and HO-1 up to 6.6- and 3.8-fold of the control, but decreased the cIAP-1 by ~0.68-fold compared with the control (Figure 4B). Green fluorescence-labelled active caspase-3 was observed and increased in HSC-3 cells after CLEFMA treatment using NucView 488 green caspase-3 dye (Figure 4C). The Western blot results indicated that, upon the treatment of HSC-3 and SCC-9 cells with CLEFMA, the expression of cIAP-1 was remarkably inhibited in HSC-3 at 1 μM (Figure 4D), while the inhibitory effect of cIAP-1 in SCC-9 was significantly reduced at 8 μM (Figure 4E). CLEFMA at 4 μM remarkably elevated the expression of HO-1 in HSC-3 cells (Figure 4D), while the levels of HO-1 were significantly increased in SCC-9 at 1 μM CLEFMA (Figure 4E). CLEFMA noticeably increased the expression HO-1 in HSC-3 (Figure 4D) and SCC-9 (Figure 4E). The inhibitory effects of cIAP-1 are more potent on HSC-3 cells compared with SCC-9 cells after CLEFMA treatment, whereas the rising effects of HO-1 are more potent on SCC-9 compared with HSC-3 cells treated with CLEFMA. The whole Western blot can be found in Figure S1. CIAP1 represses the intrinsic and extrinsic pathways of apoptosis by indirectly or directly inducing the inactivation of caspases-3, -8, and -9 [22]. Therefore, Western blot analysis was used in determining the expression of proteins belonging to the caspase family after CLEFMA treatment in HSC-3 and SCC-9 cells. The results showed that CLEFMA induced the activation of caspase-3, -8, and -9 (cleaved forms) and decreased the expression of pro-caspase-3, -8, and -9 in HSC-3 (Figure 5A) and SCC-9 (Figure 5B) cells. Subsequently, PARP was inactivated and clove by caspase-3-inducing apoptotic cell death. CLEFMA at 8 μM significantly elevated the expression of cleaved PARP, whereas the levels of PARP repressed the treatment of HSC-3 (Figure 5A) and SCC-9 (Figure 5B) cells with CLEFMA for 24 h.

### 3.5. Activation of MAPK Signalling Cascades by CLEFMA in OSCC

**Figure 6:**
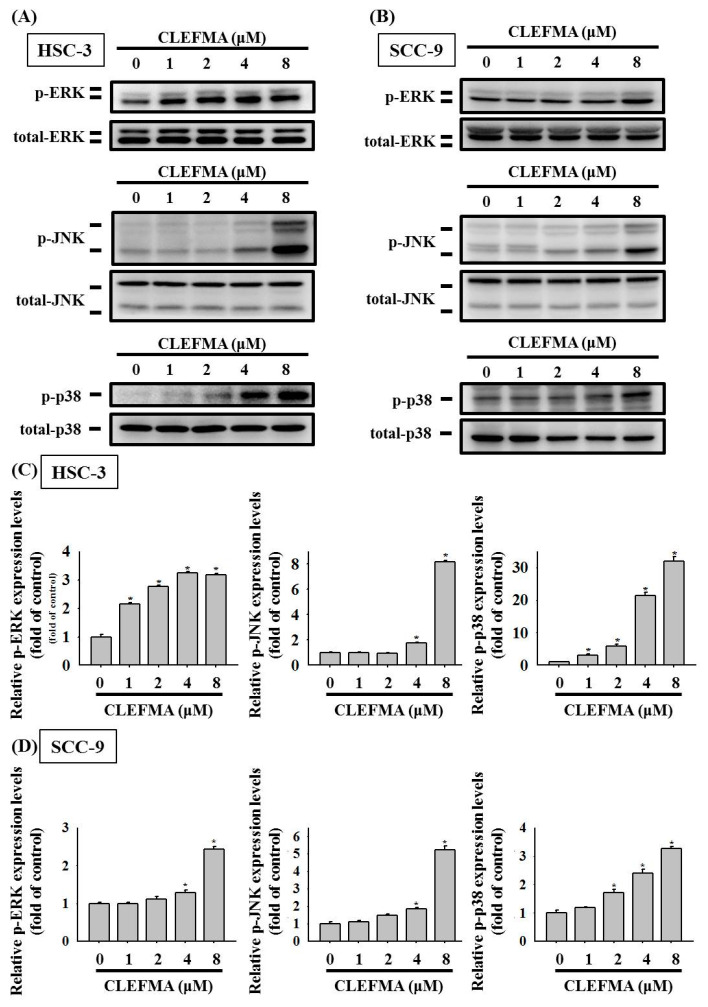
CLEFMA induced MAPK pathway activation in human OSCC. (**A**) HSC-3 and (**B**) SCC-9 cells were incubated with CLEFMA (0–8 μM). Cell lysates were subjected to Western blot assay for defining the protein level of MAPK-related pathways. The densitometry of MAPK-related protein- associated signals of (**C**) HSC-3 and (**D**) SCC-9 cells detected in (**A**,**B**), respectively. Data are presented as the mean ± SD of at least three independent experiments. * *p* < 0.05 compared with the DMSO vehicle.

The MAPK cascades play a central role in the regulation of *multiple* cellular processes, such as cell invasion, growth, metastasis, and apoptosis in cancer cells [23,24,25]. We performed Western blot analyses to determine MAPK signalling pathways stimulated by CLEFMA in OSCC cells. After treatment with CLEFMA, the levels of phosphorylation of ERK, JNK, and p38 dramatically increased in HSC-3 (Figure 6A) and SCC-9 (Figure 6B) cells. Quantification analysis indicated that the phosphorylation levels of ERK, JNK, and p38 were significantly increased after the treatment of HSC-3 cells and SCC-9 with 8 μM CLEFMA (Figure 6C,D). CLEFMA at 1 μM significantly elevated the expression of p-ERK in HSC-3 cells (Figure 6C), while the expression of p-ERK was remarkably increased in SCC-9 at 4 μM CLEFMA (Figure 6D). The expression of p-p38 was remarkably increased in HSC-3 at 1 μM CLEFMA (Figure 6C), while the expression of p-p38 was significantly up-regulated in SCC-9 at 2 μM CLEFMA (Figure 6D). Moreover, 8 μM CLEFMA treatment increased the levels of p-p38 up to 31.94- and 3.25-fold compared with the control in HSC-3 and SCC-9, respectively (Figure 6). Activation of p-ERK by CLEFMA appeared to be significantly more potent in HSC-3 (Figure 6C) compared with SCC-9 (Figure 6D), whereas the increased effects of p-p38 are more potent on HSC-3 (Figure 6C) compared with SCC-9 cells (Figure 6D) after CLEFMA treatment.

### 3.6. HO-1 Is Involved in Response to CLEFMA-Induced Caspase-Mediated Apoptosis in OSCC

**Figure 7:**
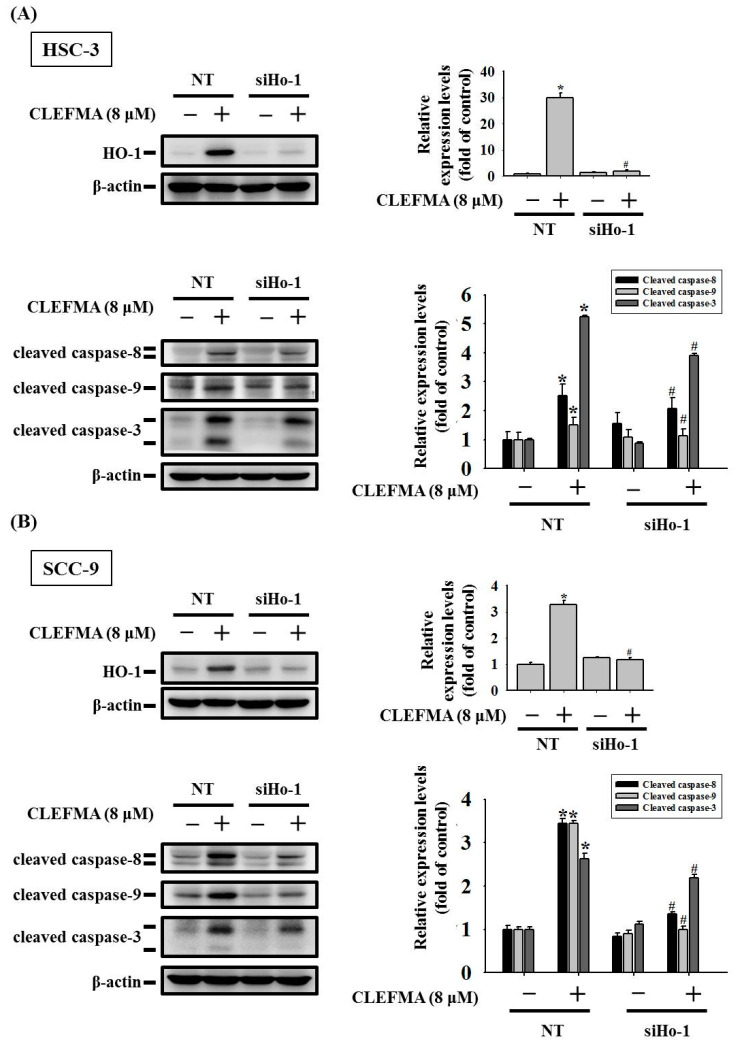
CLEFMA induced apoptotic cell death in OSCC through the up-regulation of HO-1. The protein levels of HO-1, cleaved caspase-8, cleaved caspase-9, and cleaved caspase-3 were assessed by transfection with non-targeting siRNA (NT) and HO-1 siRNA (siHo-1) in (**A**) HSC-3 and (**B**) SCC-9 cells through Western blot analysis. The densitometry of protein signals was detected (ratio to β-actin). Data are presented as the mean ± SD of at least three independent experiments. * *p* < 0.05 compared with the DMSO control transfected with NT; # *p* < 0.05 compared with the 8 μM CLEFMA treated group transfected with NT.

To further investigate the role of the up-regulated HO-1 level stimulated by CLEFMA in CLEFMA-mediated apoptotic cell death in OSCC, we silence HO-1 expression using HO-1-specific small interfering ribonucleic acid (siRNA). Western blot was performed to explore the silencing effects of HO-1-specific siRNA. We found that the transfection of HO-1-siRNA clearly reversed the up-regulation of HO-1 protein expression in CLEFMA-treated HSC-3 (Figure 7A) and SCC-9 (Figure 7B) cells. The silencing of HO-1 level dramatically reduced the CLEFMA-induced activation of caspase-8, -9, and -3 in HSC-3 (Figure 7A) and SCC-9 (Figure 7B) compared with non-targeting control siRNA-treated cells. More reductions in caspase-8, -9, and -3 by HO-1-specific siRNA are seen in SCC-9 compared with HSC-3.

### 3.7. Induction of Caspase-Mediated Apoptosis by CLEFMA Is Dependent on the Activation of p38 in OSCC

**Figure 8:**
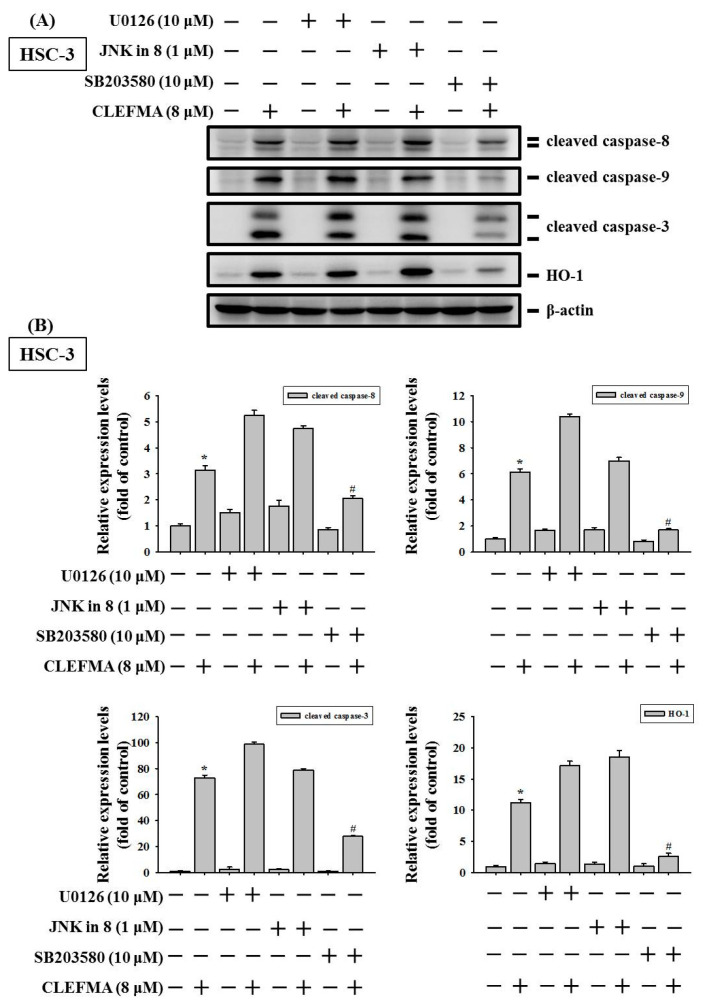

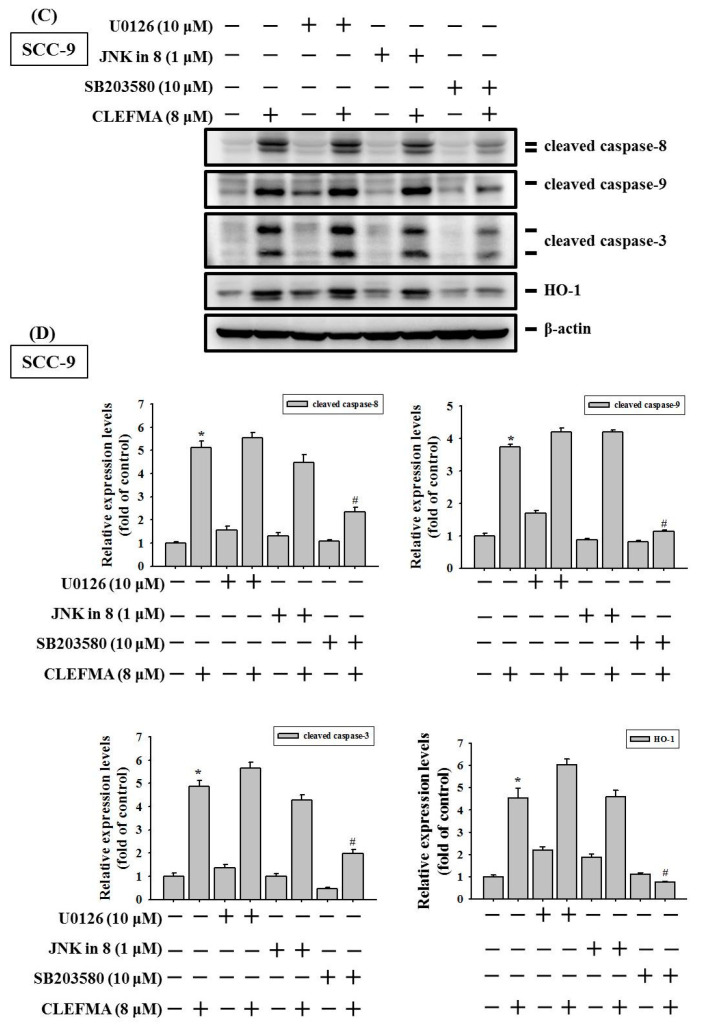
CLEFMA induced the apoptosis of human OSCC via the regulation of p38/ HO-1 signalling cascade. (**A**,**B**) HSC-3 and (**C**,**D**) SCC-9 cells were pre-treated with UO126, JNK in 8, or SB203580 for 2 h before CLEFMA (8 μM) treatment for 24 h and untreated cells. Western blot assay was conducted. The quantification data are shown in the bar graph using densitometric measurement (ratio to β-actin). * *p* < 0.05 compared with the control group; # *p* < 0.05 compared with the 8 μM CLEFMA treated group.

To further elucidate the role of MAPK signalling pathways in CLEFMA-induced apoptosis of OSCC, cells were pretreated with 10 μM U0126 (ERK inhibitor), 1 μM JNK-in-8 (JNK1/2 inhibitor), and 10 μM SB20358 (p38 inhibitor), and then 8 μM CLEFMA was added for another 24 h for Western blot analyses. SCC-9 and HSC-3 cells without CLEFMA were also analysed. The results showed that SB203580 treatment significantly rescued the CLEFMA-induced increase in the protein levels of cleaved caspase-8, -9, and -3 compared with CLEFMA alone, whereas the attenuation of p38 MAPK obviously reversed CLEFMA-induced HO-1 expression in HSC-3 (Figure 8A,B) and SCC-9 (Figure 8C,D) cells. However, ERK and JNK1/2 inhibitors had no significant effect on the recovery of CLEFMA-induced cleaved caspases and HO-1 expression (Figure 8). These findings suggested that activation of the p38 MAPK signalling cascades may increase HO-1 expression and induce apoptosis by CLEFMA in OSCC.

### 3.8. Inhibition of Tumour Growth by CLEFMA Treatment In Vivo

**Figure 9:**
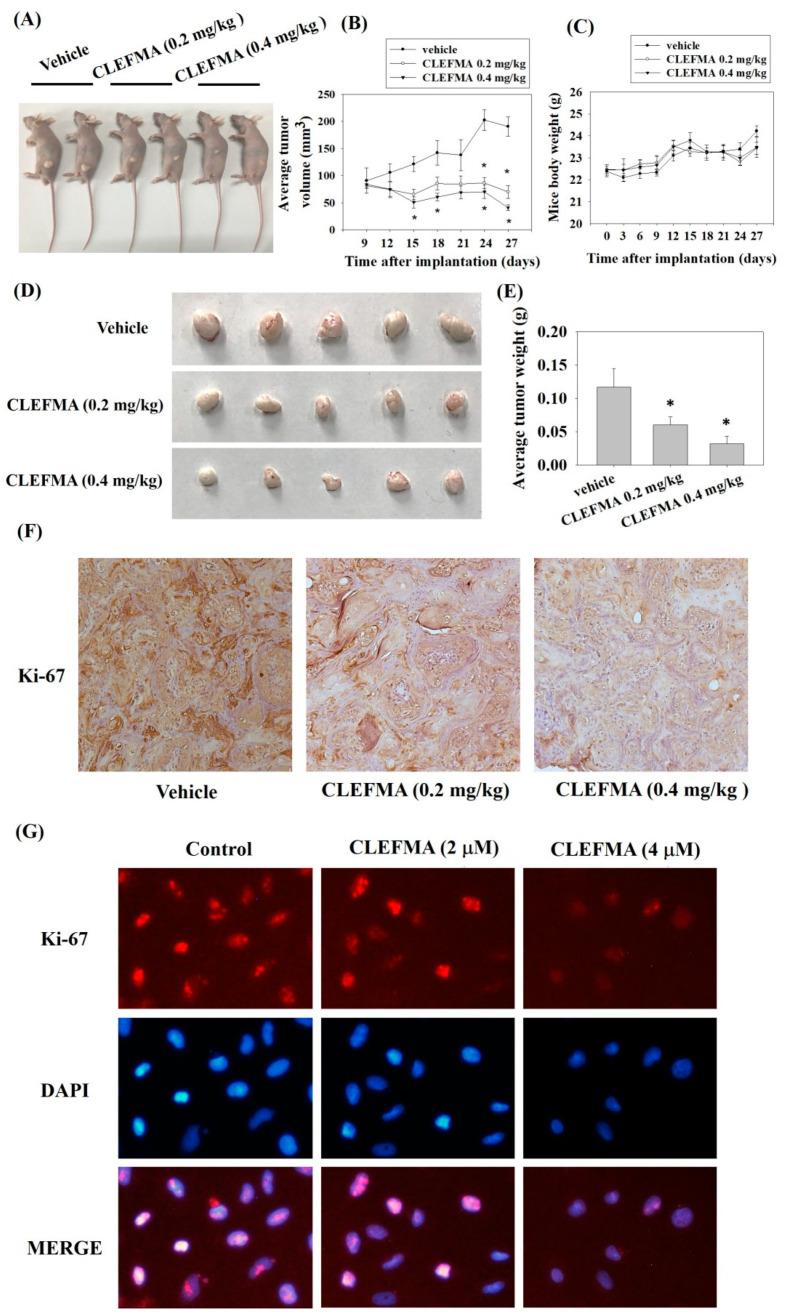
Anti-tumour effects of CLEFMA in vivo. Mice (*n* = 5) were orally administrated with either vehicle (control group) or CLEFMA (0.2 or 0.4 mg/kg) after SCC-9 cells were subcutaneously implanted. Tumour growth was then analysed. (**A**) After 27 days, the mice images were taken. (**B**) Tumour volume at each time interval during the experiment. (**C**) Average body weight of the mice during the treatment of CLEFMA. (**D**) Representative tumours isolated from mice. (**E**) Average tumour weight at the end of the treatment. (**F**) Immunohistochemistry to examine Ki-67 in SCC-9 tumours. (**G**) Immunofluorescence analysis of Ki-67 in SCC-9 cell lines. * *p* < 0.05 compared with the vehicle group.

We subcutaneously inoculated SCC-9 cells into the right flank of each nude mouse to investigate the anti-tumour effects of CLEFMA in vivo. The mice were orally administered with a vehicle (0.22% polyethylene glycol 400 sterile water solution) or CLEFMA (0.2 or 0.4 mg/kg). Animal images revealed that CLEFMA obviously decreased tumour sizes after 27 days of feeding compared with the control vehicle (Figure 9A). The mean tumour volumes remarkably decreased after 0.2 or 0.4 mg/kg CLEFMA treatment on day 27, and the volumes were 63.3% and 78.6% lower than the corresponding volumes analyzed in the vehicle-receiving animals, respectively (Figure 9B). Moreover, the average mouse body weights of the CLEFMA-treated mice and vehicle group were insignificantly affected (Figure 9C). Representative tumours were isolated from mice 27 days after subcutaneous inoculation of SCC-9 cells (Figure 9D). CLEFMA treatment obviously reduced the average tumour weights compared with the vehicle group (Figure 9E). CLEFMA treatment obviously reduced the intensity of Ki-67 (a proliferation marker) protein stain in the primary tumour (Figure 9F). Immunofluorescence analysis confirmed that CLEFMA reduced Ki-67 levels in SCC-9 cells (Figure 9G).

## 4. Discussion

Apoptosis is a form of programmed cell death that is a natural way of removing aged cells and is important to the homeostasis and development of the body. Most of the anti-tumour treatment strategies induce apoptosis and related programmed cell death networks to defeat malignant tumours [26]. Apoptosis is also one of the most studied topics in clinical oncology [27]. It can be trigged in tumourous cells through an external (extrinsic) or internal (intrinsic) cell death stimulus, which converges on the regulation of membrane blebbing, DNA fragmentation, and the caspase-mediated proteolysis of thousands of cellular proteins [27].

The activation of initiator caspases, such as caspase 9, leads to the formation of apoptosomes; this process can be negatively modulated by members of the IAP family proteins. IAP proteins, a number of signalling cascades promoting anti-apoptosis, and the cell survival effect are overexpressed in various solid and haematological malignancies [28] and associated with poor prognostic impact and 5-FU resistance in OSCC [29]. Several IAP family proteins function by binding to and inhibiting specific caspases and are critical targets for anti-cancer therapy [30]. Cellular IAP-1 (cIAP-1) bound specifically to the terminal effector cell death proteases, caspases-3 and -7, inhibited the activity of caspase-3 and -7, exerting an important influence on resistance to apoptosis in many types of cancer and leading to cell survival. Recent research has shown that the high mRNA level of cIAP-1 is associated with distant organ metastasis in patients with breast cancer [31]. In the present study, we demonstrated that CLEFMA up-regulated the early and late phase of apoptotic cell population by flow cytometry. CLEFMA treatment significantly increased the protein levels of active caspase-8, -9, and -3, whereas the expression of cleaved-PARP was elevated in OSCC. In addition, cIAP-1 level was remarkably reduced after 24 h of CLEFMA treatment in HSC-3 and SCC-9 cells. Nuclear factor κB (NF-κB) is an important transcription factor regulating the gene expression of cIAP [32]. The activation of NF-κB in OSCC induces cell growth, cell survival, and cisplatin resistance [33]. Overall, CLEFMA might improve the therapeutic effects against OSCC by targeting the NF-κB/cIAP-1 signalling pathway, and this hypothesis should be further investigated in the future.

In addition to cIAP-1 signalling, CLEFMA obviously elicits an increase in the HO-1 level in a proteome profiler human apoptosis array in OSCC cells. The biological properties of HO-1 metabolise heme into iron, carbon monoxide (CO), and biliverdin or bilirubin, as well as the cytoprotective capabilities of HO-1 against a wide range of cellular stressors and pro-inflammatory mediators and cytokines, have been demonstrated in many studies. An increase in HO-1 expression suppresses inflammation and carcinogenesis. Curcumin can decrease the growth of human breast cancer cells by raising HO-1 expression, which elicits CO expression and results in attenuation of heat shock protein 90 activities [34]. HO-1 expression is up-regulated in a number of cancer types and contributes to cancer progression [35]. A high HO-1 expression level in bone marrow stromal cells can trigger multiple resistances by targeting the JAK2/STAT3 pathway in myeloma [36]. These findings suggested the dual role of HO-1 in the progression of tumours [37]. As for the role of HO-1 in OSCC, low HO-1 expression levels were remarkably correlated with a rising risk of lymph node metastasis in patients with tongue carcinomas [38]. Moreover, the Nrf-2 transcription factor maintains cellular homeostasis associated with oxidative stress and regulates the expression of HO-1 [39]. The data presented here demonstrated that HO-1 expression was obviously up-regulated in OSCC cells after treatment with CLEFMA. The silencing of HO-1 expression remarkably attenuated CLEFMA-induced caspase-dependent cell death in OSCC, suggesting that CLEFMA-induced HO-1 production plays a positive role in the proapoptotic activities of CLEFMA in OSCC. In the future, the relationship between CO and HO-1 of OSCC and the level of HO-1 by further inducing the activation and nuclear translocation of Nrf2 following the CLEFMA treatment are worthy of further investigation.

We next explored CLEFMA-mediated upstream signal transduction in modulated HO-1 expression. A recent report showed that induction of HO-1 expression is closely linked to kinase signalling pathways, including ERK, JNK, and p38. The MAPK have long been implicated the regulation of cell proliferation, cell cycle, cell migration, and apoptosis in many cancer cells [3,4,40]. Increasing evidence has indicated that high-expression and activation of the MAPK signalling pathway in cancer may lead to abnormal cell division and apoptosis resistance [41]. In the current study, the level of p-p38 was significantly increased in HSC-3 at 1 μM CLEFMA, while the expression of p-p38 was remarkably up-regulated in SCC-9 at 2 μM CLEFMA. The phosphorylation levels of p38 were increased up to 31.94- and 3.25-fold compared with the control in HSC-3 and SCC-9 after 8 μM CLEFMA treatment, respectively (Figure 6). Activation of p-p38 by CLEFMA appeared to be significantly more potent in HSC-3 compared with SCC-9. Western blot analysis also confirmed that p38 inhibitor SB203580 appeared to be more effective in HSC-3 than SCC-9 in reversing the effect of CLEFMA through the p38 pathway. HSC-3 (tumours of metastatic lymph nodes) showed a higher invasive capacity and metastatic phenotype compared with SCC-9 (primary OSCC). These findings suggest that the pro-apoptotic and growth inhibitory effects of CLEFMA are more potent on high-metastatic HSC-3 compared with SCC-9 cells. Accumulating evidence indicates that the stimulation of caspase-8, -9, and -3 activation by CLEFMA was reversed by the p38 inhibitor, SB203580, suggesting that p38 MAPK activity is essential to the pro-apoptotic ability of CLEFMA. Moreover, the inhibition of p38 MAPK obviously reversed CLEFMA-induced HO-1 expression in HSC-3 and SCC-9 cells. Overall, these results suggested that the activation of the p38 MAPK signalling cascades may increase HO-1 expression and caspase-dependent cell death induced by CLEFMA in OSCC.

It is well documented that curcumin acts against cancer by altering multiple cellular targets and a broad set of signal transduction pathways, including up-regulation of toxic intracellular ROS release, induction of apoptotic cell death, modulation of microRNAs, cell cycle arrest, and regulation of various protein kinases cascades as well as NF-ĸB and p53 signaling pathways [42]. However, curcumin displays limited bioavailability, poor absorption, and rapid systemic elimination, which restrict its efficacy and contribute to the low levels of curcumin in plasma [43]. Structural analogues of curcumin, including CLEFMA, have now been demonstrated that have various anti-cancer efficacy and improved stability and bioavailability [44,45]. Current available chemotherapy agents show limited effectiveness, accompanied by deleterious side effects for patients with cancer. In this study, human gingival epithelial SG cells were used to demonstrate that CLEFMA did not cause cytotoxic effects on non-malignant gingival epithelial cells. CLEFMA could improve the inhibitory effects of paclitaxel on the cell viability of SCC-9 cells.

## 5. Conclusions

In summary, we demonstrated the anti-cancer action of CLEFMA in association with the induction of HO-1 and apoptotic cell death in OSCC. The findings from the current study indicated that CLEFMA increased HO-1 production via the activation of p38 signalling and subsequently activated caspase-8, -9, and -3, which are critical for the anti-cancer action in HSC-3 and SCC-9 cells. In addition, the oral administration of CLEFMA effectively inhibited the growth of SCC-9-derived xenograft tumour immunodeficient nude mice. Accumulating evidence has suggested that CLEFMA is a potential adjuvant therapeutic agent for treating OSCC.

## Data Availability

The datasets generated for this study are available upon request to the corresponding authors.

## Supplementary Materials

The following supporting information can be downloaded at https://www.mdpi.com/article/10.3390/cancers14225519/s1. Figure S1: The whole Western blot figures.
