# Supplementary figures and images for: CLEFMA Induces the Apoptosis of Oral Squamous Carcinoma Cells through the Regulation of the P38/HO-1 Signalling Pathway
Source: Cancers (Basel). 2022 Nov 10;14(22):5519. doi: 10.3390/cancers14225519 (PMC9688613; doi:10.3390/cancers14225519)

Figure S1: The whole Western blot figures.

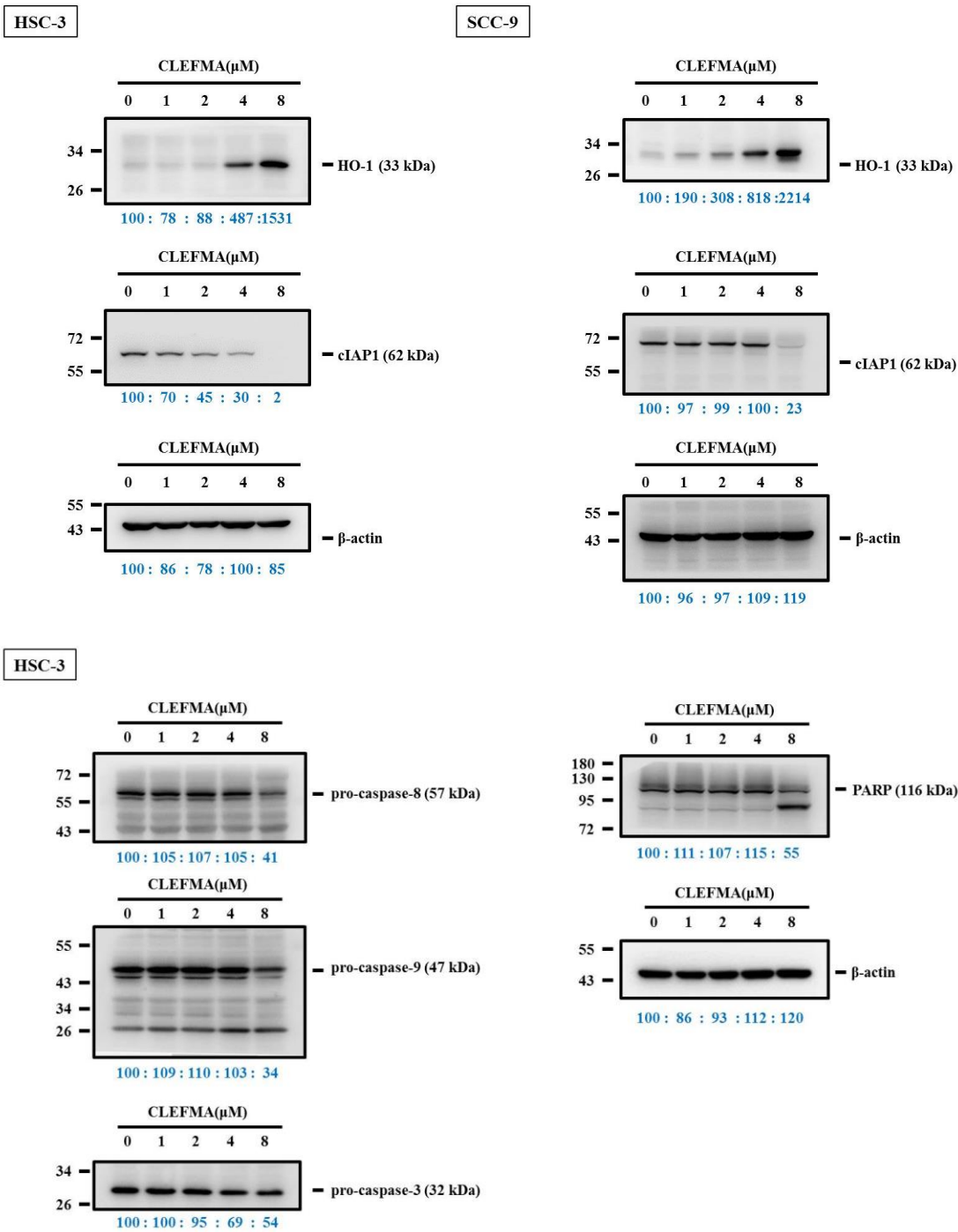

**HSC-3**

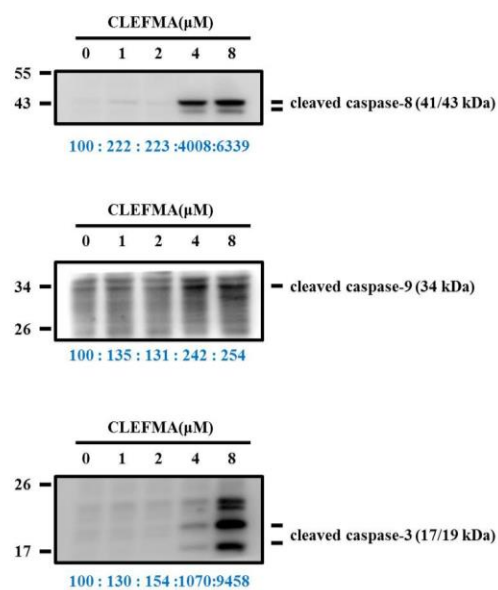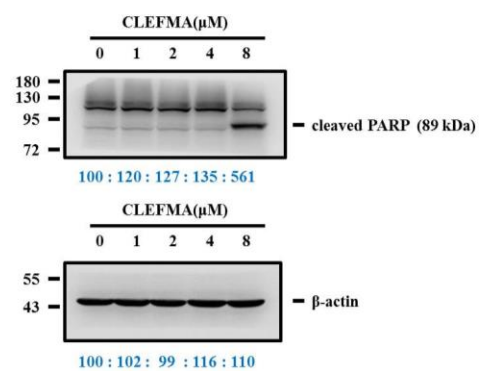

**SCC-9**

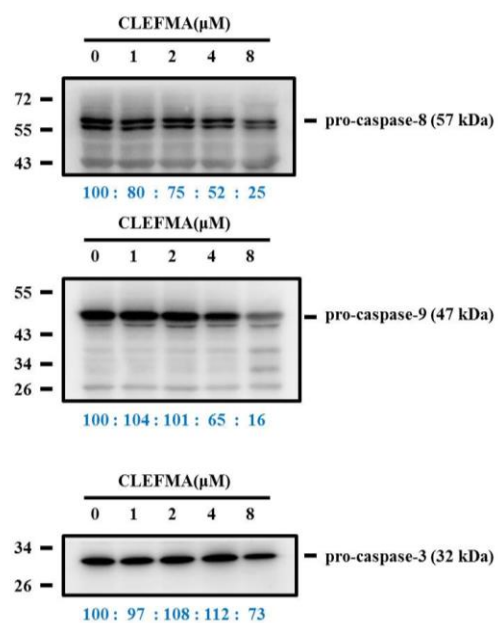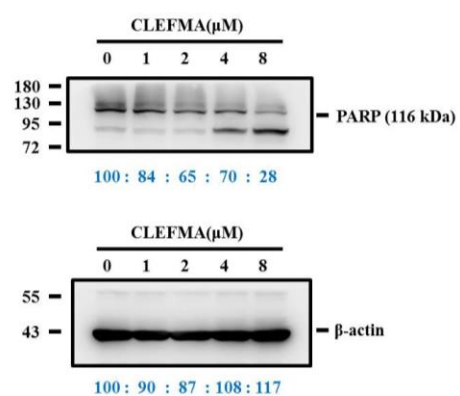

SCC-9

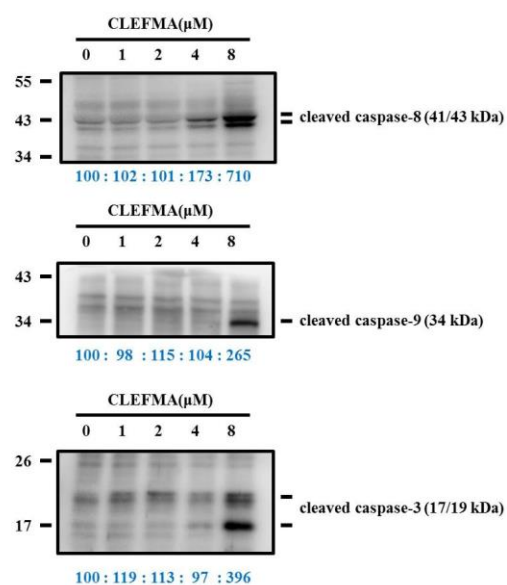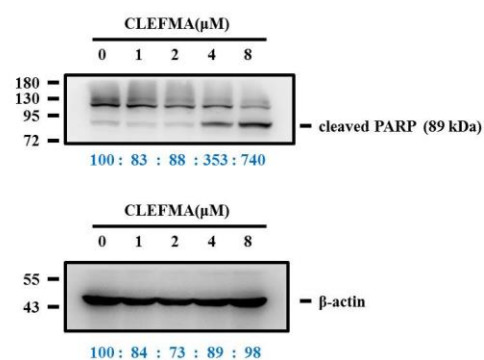

HSC-3

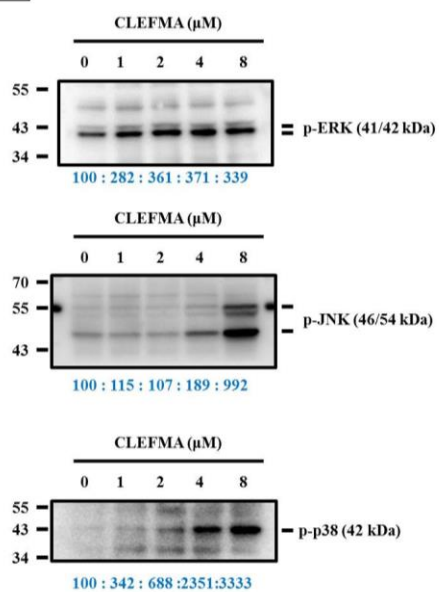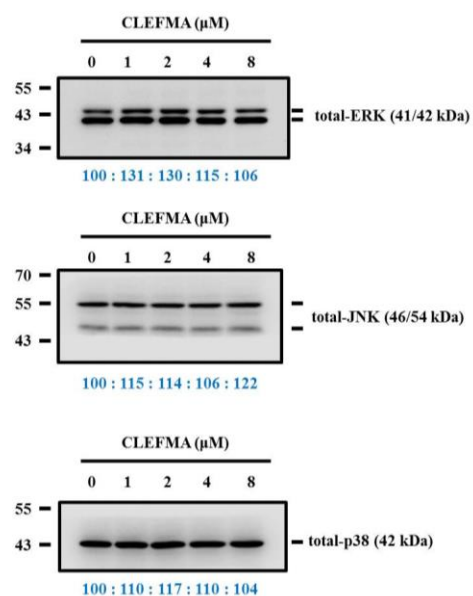

SCC-9

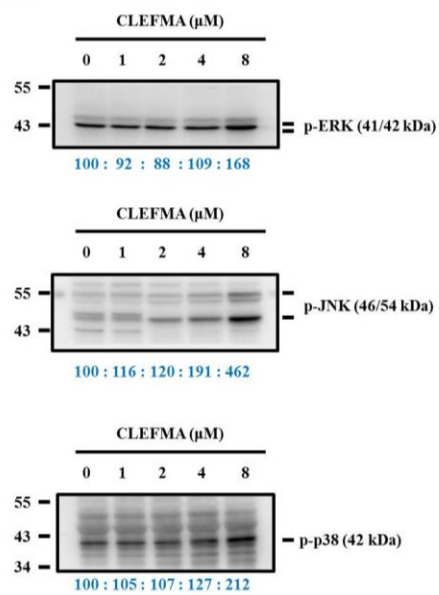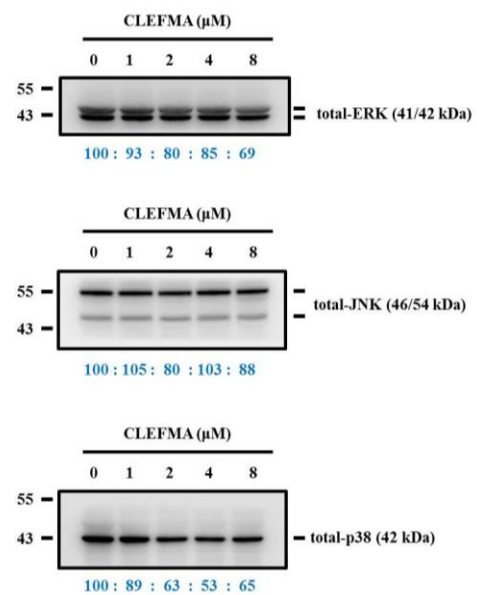

HSC-3

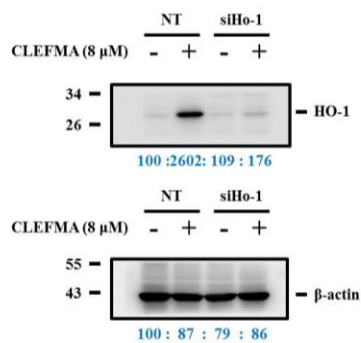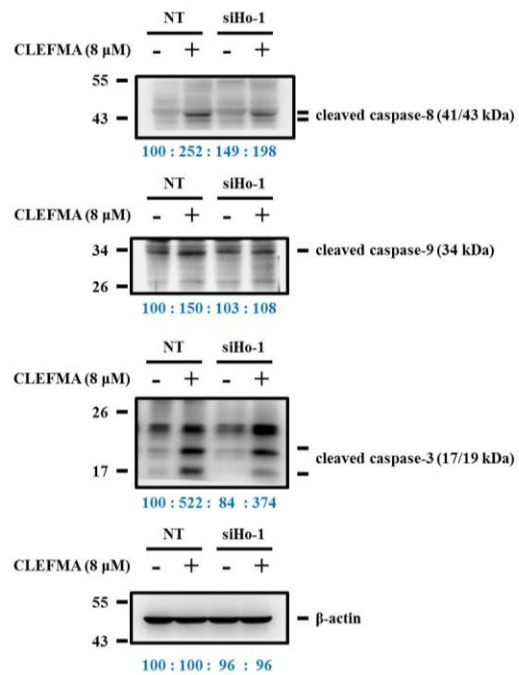

# SCC-9

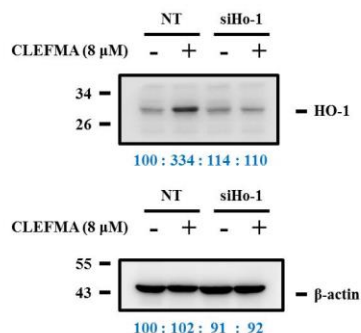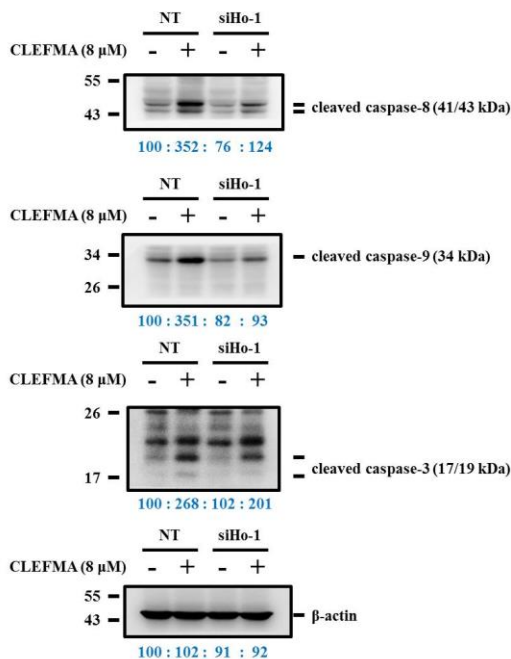

# HSC-3

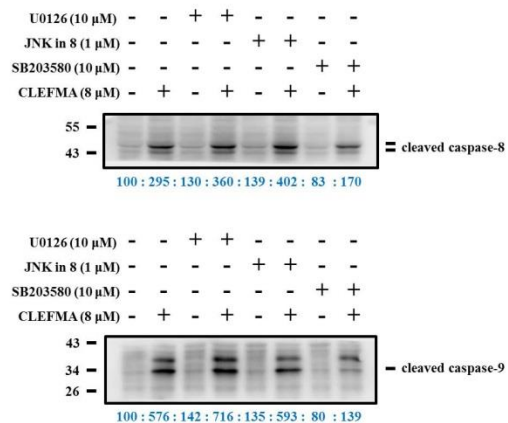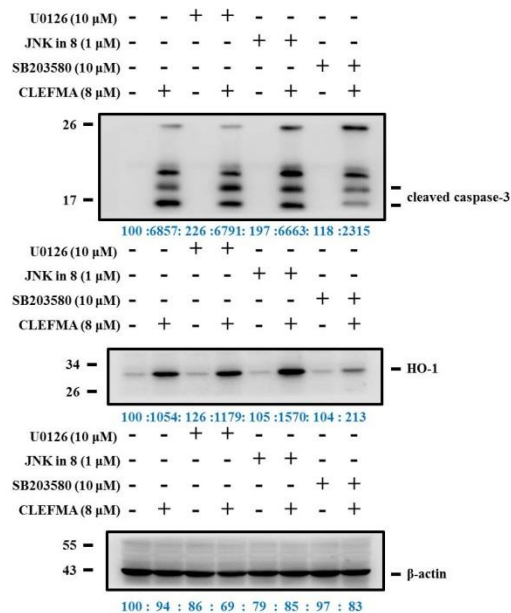

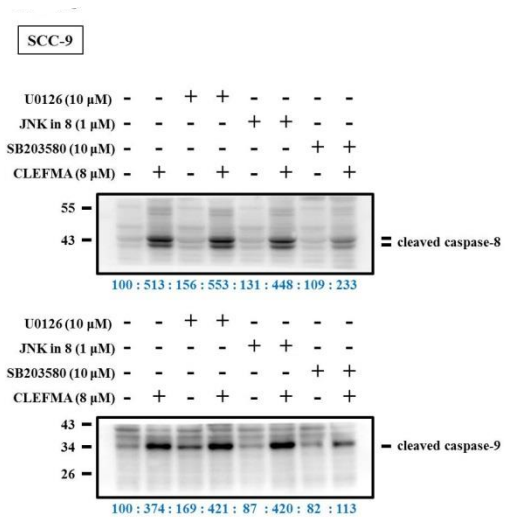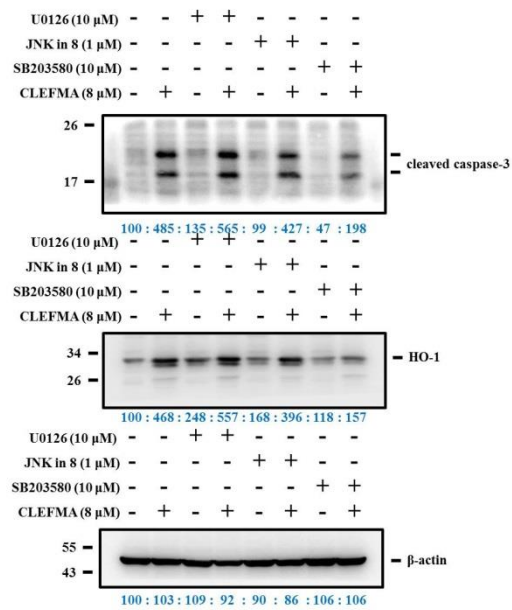

Supplement: Supplementary file 1 [file cancers-14-05519-s001.zip › cancers-1957018-supplementary.pdf]
